# Chitosan-Coated PLGA Nanoparticles Loaded with *Peganum harmala* Alkaloids with Promising Antibacterial and Wound Healing Activities

**DOI:** 10.3390/nano11092438

**Published:** 2021-09-18

**Authors:** Hassan Mohamed El-Said Azzazy, Sherif Ashraf Fahmy, Noha Khalil Mahdy, Meselhy Ragab Meselhy, Udo Bakowsky

**Affiliations:** 1 Department of Chemistry, School of Sciences & Engineering, The American University in Cairo, AUC Avenue, New Cairo 11835, Egypt; sheriffahmy@aucegypt.edu (S.A.F.); noha.khalil@aucegypt.edu (N.K.M.); 2 Department of Pharmacognosy, Faculty of Pharmacy, Cairo University, Kasr El-Aini Street, Cairo 11562, Egypt; meselhy.meselhy@pharma.cu.edu.eg; 3 Department of Pharmaceutics and Biopharmaceutics, University of Marburg, Robert-Koch-Str. 4, 35037 Marburg, Germany

**Keywords:** chitosan-coated PLGA nanoparticles, *Peganum harmala* alkaloids, Box–Behnken design, antibacterial, wound healing

## Abstract

Wound healing is a major healthcare concern, and complicated wounds may lead to severe outcomes such as septicemia and amputations. To date, management choices are limited, which warrants the search for new potent wound healing agents. Natural products loaded in poly (lactic-co-glycolic acid) (PLGA) coated with chitosan (CS) constitute a promising antibacterial wound healing formulation. In this work, harmala alkaloid-rich fraction (HARF) loaded into PLGA nanoparticles coated with chitosan (H/CS/PLGA NPs) were designed using the emulsion-solvent evaporation method. Optimization of the formulation variables (HARF: PLGA and CS: PLGA weight ratios, sonication time) was performed using the 3^3^ Box–Behnken design (BBD). The optimal NPs were characterized using transmission electron microscopy (TEM) and Attenuated Total Reflection Fourier-Transformed Infrared Spectroscopy (ATR-FTIR). The prepared NPs had an average particle size of 202.27 ± 2.44 nm, a PDI of 0.23 ± 0.01, a zeta potential of 9.22 ± 0.94 mV, and an entrapment efficiency of 86.77 ± 4.18%. In vitro drug release experiments showed a biphasic pattern where an initial burst of 82.50 ± 0.20% took place in the first 2 h, which increased to 87.50 ± 0.50% over 72 h. The designed optimal H/CS/PLGA NPs exerted high antibacterial activity against *Staphylococcus aureus* and *Escherichia coli* (MIC of 0.125 and 0.06 mg/mL, respectively) compared to unloaded HARF (MIC of 0.50 mg/mL). The prepared nanoparticles were found to be biocompatible when tested on human skin fibroblasts. Moreover, the wound closure percentage after 24 h of applying H/CS/PLGA NPs was found to be 94.4 ± 8.0%, compared to free HARF and blank NPs (68.20 ± 5.10 and 50.50 ± 9.40%, respectively). In conclusion, the three components of the developed nanoformulation (PLGA, chitosan, and HARF) have synergistic antibacterial and wound healing properties for the management of infected wounds.

## 1. Introduction

Wound healing represents a major healthcare challenge. Considerable efforts have been exerted to develop effective wound healing products which minimize scar formation. The wound healing process consists of four phases: hemostasis, inflammation, proliferation (via the release of cytokines), and maturation with full wound closure [1,2]. One of the significant issues that can delay wound healing/closure is infection with Gram-positive and/or Gram-negative bacteria such as *Staphylococcus aureus* and *Escherichia coli,* respectively. These pathogens can infect deep skin tissues in chronic wounds, leading to the perturbation of the physiological wound healing process [3,4]. Natural products such as plant extracts have drawn much attention in the remedy of many diseases [5,6,7]. *Peganum harmala* L. (*Zygophyllaceae* family) is a plant that grows wildly in the Middle East and North Africa. Its seeds have been used as an antibacterial, antifungal, antipruritic, and antioxidant, and also in wound healing [8,9]. The seeds of *P. harmala* are rich in therapeutically active β-carboline and quinazoline alkaloids (harmala alkaloids). These alkaloids, including harmine, harmol, harmaline, harmane, harmalol, and peganine, are responsible for the therapeutic activities of the harmala seeds [8,9]. The promising wound-healing ability of *P. haramala* extract was reported. It showed the capacity to decrease the wound epithelial gap and increase the collagen and fibroblasts in the wound microenvironment, resulting in a rapid and effective healing process [10]. Despite the current clinical use of many wound healing agents, they still suffer from being sensitive to the wound microenvironment, leading to unsatisfactory structural and functional clinical outcomes [11].

Consequently, several delivery systems, including liposomes, macrocycles, and polymeric nanoparticles, were reported to significantly enhance the therapeutic effects of various natural and synthetic drugs [12,13,14,15,16,17,18]. Some natural and synthetic polymers were found to have healing properties by improving proliferation and increasing the cell count. Moreover, they are biodegradable and biocompatible while maintaining exceptional physicochemical and mechanical properties [19,20]. For instance, chitosan (CS) possesses antibacterial, bio-adhesive, and hemostatic properties, making it a perfect candidate in wound healing systems. CS was reported to promote wound healing by enhancing the migration of fibroblasts and collagen deposition in the wound area [21,22,23]. Furthermore, CS is a perfect carrier for different wound healing agents that protect these agents from side reactions in the wound microenvironment that may cause their deactivation while simultaneously increasing their absorption and targeting [24,25]. Poly (lactic-co-glycolic acid) (PLGA), a copolymer of polylactic and polyglycolic acids, is an example of synthetic wound healing polymers. PLGA, an FDA-approved polymer, is considered biodegradable and was found to act as a wound-healing agent. PLGA is a source of lactate that accelerates reparative angiogenesis and, hence, promotes rapid wound healing [26,27]. Furthermore, PLGA is an outstanding carrier for different wound healing agents because of its ability to adhere to the wound surface while releasing the loaded drug in a controlled manner [28].

In the present work, H/CS/PLGA NPs were prepared using the emulsion-solvent evaporation method. Optimization of the formulation variables was performed using a 3^3^ Box–Behnken surface response design (BBD). The factors nominated for the experimental design included the HARF:PLGA weight ratio, the CS:PLGA weight ratio, and the sonication time. The selected responses to judge the effects of the factors and their interactions were the particle size, the PDI, the zeta potential, and the entrapment efficiency. Then, the optimized formulation was prepared and validated in terms of the four responses. The optimal NPs were characterized by transmission electron microscopy (TEM) and Attenuated Total Reflection Fourier-Transformed Infrared Spectroscopy (ATR-FTIR). Additionally, the release of HARF from H/CS/PLGA NPs was assessed, and the release profile was obtained. Finally, the antimicrobial and wound healing properties of H/CS/PLGA NPs were studied, and their cytotoxicity was evaluated.

## 2. Results and Discussion

### 2.1. Formulation Optimization by Three-Factor, Three-Level Box–Behnken Response Surface Design (3^3^ BBD)

To find the best model to use with the highest adjusted and prediction R^2^, the responses of particle size (PS, Y1), zeta potential (ZP, Y2), polydispersity index (PDI, Y3), and entrapment efficiency (EE%, Y4) were fitted individually to linear, two-factor interaction (2FI), and quadratic models. Analysis of Variance (ANOVA) testing was carried out at a *p*-value < 0.05. Non-significant model terms were eliminated to reach a higher prediction R^2^. As presented in Table 1, the quadratic model was chosen for the first and second responses (PS and PDI). The linear model was selected for the third response (ZP), and the 2FI model was chosen for the fourth response (EE%). The final equations related to different factors and interactions for the responses were generated after model reduction, via Design-Expert Version 12.0.1.0 (Stat-Ease Inc., Suite 480, Minneapolis, MN, USA) in terms of coded variables. The four equations were as follows:

$$\begin{array}{c} PS=199.08-0.8063A+3.89B-0.1721C+2.19AB+1.38AC+9.32BC+7.48A^{2}-1.57B^{2}-6.03C^{2}\\ PDI=0.1239-0.0144A+0.0006B-0.0013C+0.0212AB+0.0718AC+0.0057BC+0.0416A^{2}-0.0144B^{2}\;+0.0048C^{2}\\ ZP=8.14+0.48A+3.72B-0.71C\\ EE\%=50.87-12.02A+7.52B+1.49C-17.32AB-11.51AC+2.72BC\; \end{array}$$

#### 2.1.1. Influence of the Independent Factors on Particle Size (PS)

**Figure 1:**
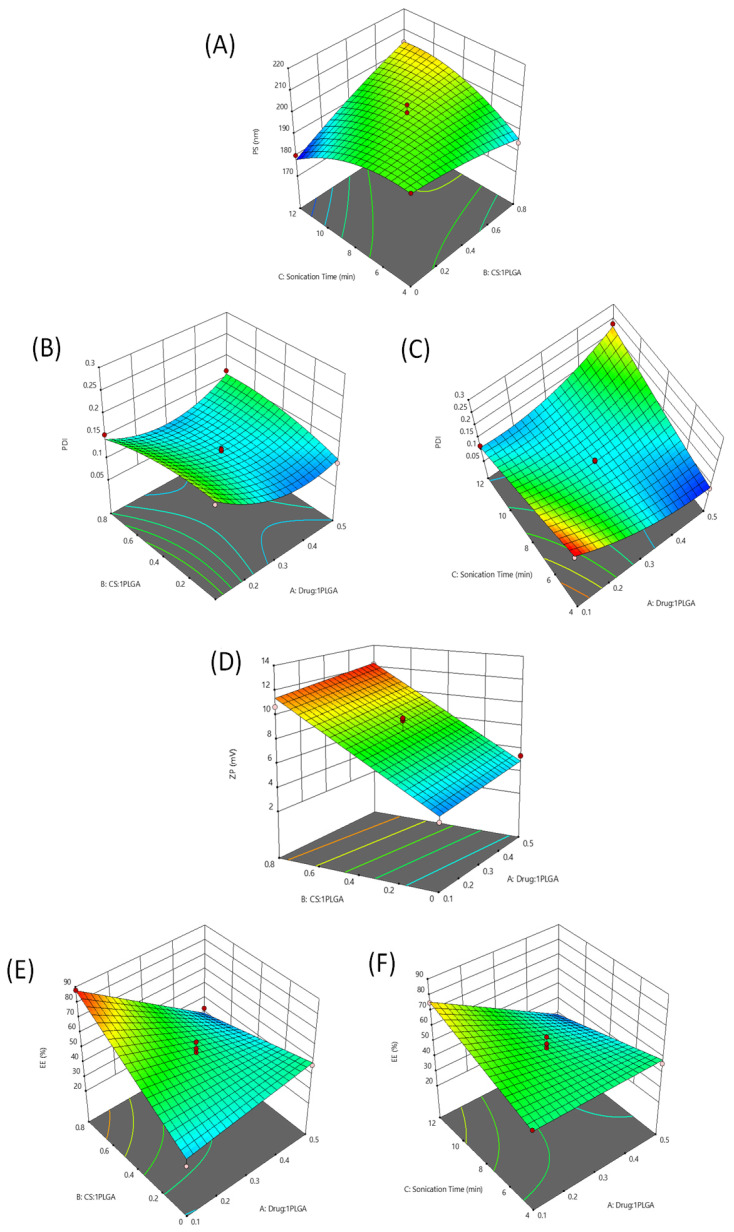
Three-dimensional surface plots for the main effects and interactions of the HARF: PLGA ratio, the CS: PLGA ratio, and the sonication time on PS (**A**), PDI (**B**,**C**), ZP(**D**), and EE % (**E**,**F**).

The small particle size of the prepared chitosan-coated PLGA nanoparticles loaded with harmala alkaloid-rich fraction (H/CS/PLGA/NPs) was shown to improve cell membrane adhesion and penetration, leading to an enhanced therapeutic efficiency [29]. The PS of the prepared H/CS/PLGA NPs ranged from 180.12 up to 214.10 nm (as shown in Table S1), suggesting the capability to produce small particle sizes. ANOVA analysis showed that factors B (CS: PLGA ratio) and C (sonication time) significantly affected PS (both having *p* < 0.0001). Increasing factors B and C to a specific limit led to a significant increase in particle size, as presented in Figure 1A. This increase in particle size may be attributed to the increase in the thickness of chitosan coating around the PLGA nanoparticles [30]. The three independent variables were the HARF:PLGA weight ratio (A), the CS:PLGA weight ratio (B), and the sonication time (C).

#### 2.1.2. Influence of the Independent Factors on PDI

The PDI value determines the NP homogeneity, with a PDI < 0.3 considered optimal. The PDI of the prepared H/CS/PLGA NPs ranged from 0.08 to 0.25 (Table S1) [31]. The ANOVA analysis showed that the PDI of the prepared nanoparticles was significantly affected by factor A (HARF:PLGA ratio), which showed interactions with the other two factors. Each of these interactions is presented in the 3D surface plots (Figure 1B,C). The PDI decreased significantly (*p* < 0.05) with the increasing of factor A (HARF:PLGA weight ratio). This may have been due to the increment of the inclusion of hydrophobic HARF, which enhanced the compactness of PLGA, leading to rearrangement and an improvement in particle size control. Therefore, factor B may be involved in the AB interaction (*p* < 0.05), leading to a reduction in PDI due to its significant positive effect on entrapment efficiency (discussed in Section 2.1.4). The increasing of the sonication time decreased the nanoparticles’ particle sizes, which facilitated the improvement of PDI values, resulting in the AC interaction (*p* < 0.0001) [32].

#### 2.1.3. Influence of the Independent Factors on ZP

The stability of NPs is directly proportional to the zeta potential value. Nanoparticles showing higher zeta potential possess higher charges, leading to stronger repulsive forces between the particles, which hinder aggregation and sedimentation of the prepared nanoparticles [33]. The positive ZP values of the prepared H/CS/PLGA NPs, ranging from 2.63 up to 12.33 mV (Table S1), are attributed to the positively charged amino groups found in CS, which coats the NPs. The obtained positively charged nanoparticles would be able to cross cell membranes efficiently [34]. The ANOVA analysis of this response showed that factor B significantly affected the ZP of the nanoparticles (*p* < 0.0001). The planar 3D surface graph presents this effect in Figure 1D. Increasing factor B significantly increased the zeta potential value. This can be attributed to the charged protonated amine groups on the surface of the chitosan-coated PLGA nanoparticles [35]. 

#### 2.1.4. Influence of the Independent Factors on Entrapment Efficiency (EE %)

The EE % of the prepared H/CS/PLGA NPs ranged from 24.89% to 87.84% (Table S1). The ANOVA for this response showed that it was significantly influenced by factors A and B, both separately (*p* < 0.0001 and *p* < 0.001; respectively) as well as together (*p* < 0.0001). The 3D surface plot in Figure 1E presents these effects. The decrease in entrapment efficiency (%) with the increasing of the drug:PLGA ratio is attributed to the saturation of the nanoparticles with the loaded drug. On the other hand, the increase in the entrapment efficiency (%) with the increasing of the CS:PLGA ratio could be due to the reduced leakage of the entrapped materials [36]. Additionally, a second interaction appeared between factors A and C (*p* < 0.001), suggesting that the increase in sonication time renders the negative effect of factor A more prominent, as presented in Figure 1F. This is due to the enhanced partitioning of the drug in the aqueous phase with sonication since ultrasonication leads to the diffusion of the active constituents across different phases [37].

### 2.2. Characterization of the Optimized H/CS/PLGA NPs

The optimized formulation was selected using the Design-Expert 12^®^ software (Stat-Ease Inc., Minneapolis, MN, USA), with an overall desirability of 0.817, after applying PS, PDI, ZP, and EE % constraints. The particle size criteria were in range, as all the prepared formulations were acceptable regarding these responses. The constraints applied on the PDI, zeta potential, and entrapment efficiency %, were minimized, maximized, and maximized with a lower limit of 80%, respectively (Table 2). The suggested optimum formulation was prepared and characterized for evaluation of the optimization process. The observed PS, PDI, ZP, and EE % between the low and high confidence intervals of the predicted values (Table 1) confirmed the validity of the optimization process.

**Figure 2:**
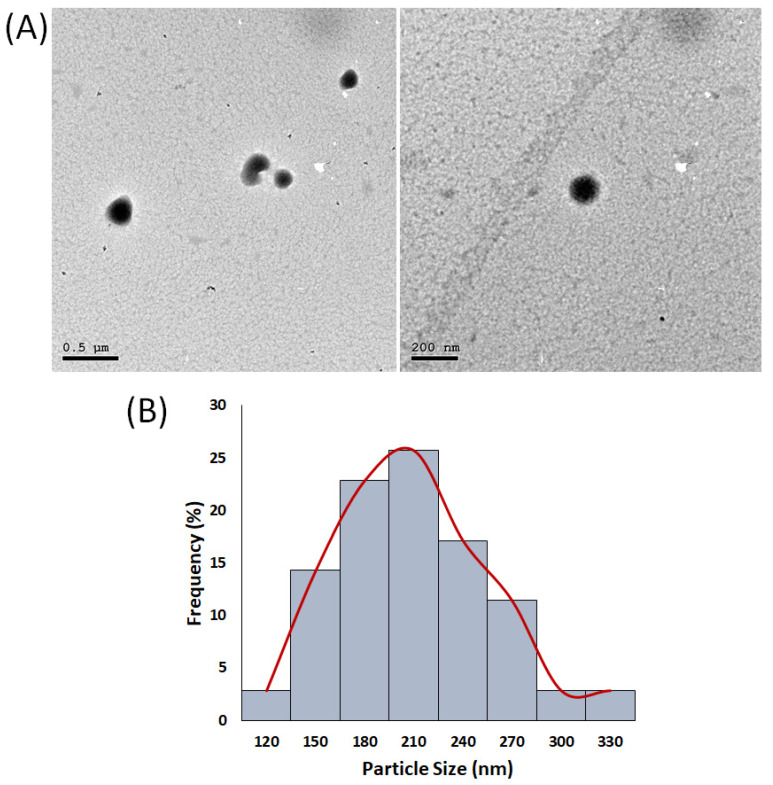
(**A**) Transmission electron microscopy (TEM) images of the optimal H/CS/PLGA NPs formulation. (**B**) Particle size (nm) histogram of the optimal H/CS/PLGA NPs formulation, produced using the image processing program Image J (NIH, Bethesda, MD, USA).

Transmission electron microscopy (TEM) analysis was employed to determine the particles’ shape and average size. As illustrated in Figure 2A, the NPs displayed a spherical shape, with some being agglomerated, which agreed with previous reports on CS and PLGA polymeric NPs [5,38]. The mean average size of the optimal H/CS/PLGA NPs formulation was determined by employing the image processing program Image J (NIH, Bethesda, MD, USA) and was found to be 194 ± 47 (Figure 2B), which is close to the one obtained from the Zetasizer (202.27 ± 2.44 nm).

### 2.3. ATR-FTIR Analysis of the Optimal H/CS/PLGA NPs Formulation

**Figure 3:**
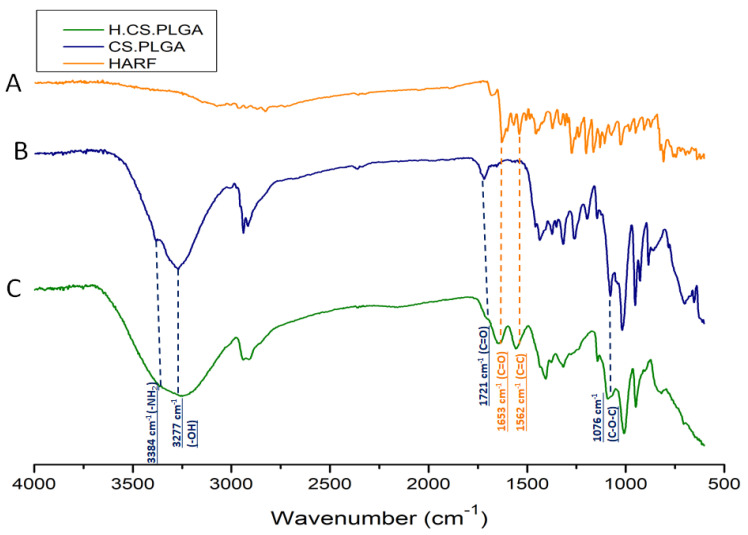
ATR-FTIR spectra of HARF (**A**), blank formulation (CS/PLGA NPs; (**B**)), and optimized H/CS/PLGA NPs formulation (**C**).

ATR-FTIR analysis of the H/CS/PLGA NPs was conducted to verify the chemical composition of the NPs. ATR-FTIR data revealed bands that can be found either in the spectrum of HARF or that of a blank formulation (CS/PLGA NPs), as presented in Figure 3. The ATR-FTIR spectrum of HARF showed characteristic peaks at 1562 and 1653 cm^−1^, which may correspond to the stretching vibrations of the alkene (C=C bond) and carboxylic (C=O bond) groups, respectively [39]. On the other hand, the spectrum of the blank formulation showed characteristic peaks of PLGA at 1076 and 1721 cm^−1^, which can be attributed to the stretching vibrations of the C–O–C and C=O bonds, respectively. In addition, there are characteristic peaks for chitosan at 3277 and 3384 cm^−1^, which may be attributed to the stretching vibrations of the –OH and –NH_2_ groups, respectively [40]. The major bands of HARF and blank formulation were found in the spectrum of H/CS/PLGA NPs, with no noticeable shift, suggesting that the entrapment of HARF was a physical entrapment [41].

### 2.4. In Vitro Release Study for the Optimal H/CS/PLGA NPs Formulation

**Figure 4:**
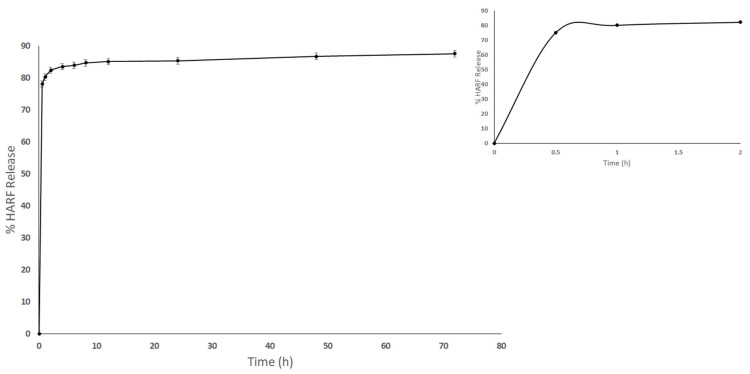
Time-dependent release profiles of HARF from the optimal H/CS/PLGA NPs, at 37 °C, into phosphate buffer media (the first 2 h are marked in the insert).

Figure 4 depicts the release profile of HARF from the H/CS/PLGA NPs into a phosphate buffer medium. The release profile showed an initial release of 82.50 ± 0.20% in the first 2 h, which increased to 87.50 ± 0.50% over 72 h. These findings agree very well with previous reports [42,43]. The sustained release behavior is attributed to the diffusion of the encapsulated HARF from the polymeric matrix after the erosion and hydrolytic cleavage of PLGA. Furthermore, the CS decorating the PLGA NPs surface plays a vital role in achieving a prolonged release behavior, which can increase the healing effect of HARF on the wound area by prolonging the contact time with skin cells [40,42,43]. 

### 2.5. Antimicrobial Assay for the Optimal H/CS/PLGA NPs Formulation

One critical issue that can delay the physiological wound healing process and might cause complications is infection with Gram-positive and/or Gram-negative bacteria such as *Staphylococcus aureus* and *Escherichia coli,* respectively [3,4]. Thus, it is warranted to use healing agents with bactericidal ability that do not interfere the fibroblast migration. The antimicrobial activities of the free HARF, CS/PLGA blank NPs, and designed H/CS/PLGA NPs were evaluated against *S. aureus* and *E. coli* utilizing the broth macrodilution method. H/CS/PLGA NPs revealed the highest antibacterial activity against *S. aureus* and *E. coli*, with MIC values of 0.125 and 0.06 mg/mL, respectively, compared to free HARF (MIC of 0.5 mg/mL) and CS/PLGA blank NPs (MIC of 0.18 mg/mL), as presented in Table 3. The nanoparticles were shown to have decomposed in the wound environment, releasing the loaded HARF, which was rich with β-carboline and quinazoline alkaloids (harmine, harmol, harmaline, harmane, harmalol, and peganine) [8,9]. These alkaloids exerted their antibacterial action via the intercalation with bacterial DNA. The pronounced antibacterial activities of the prepared NPs would disinfect the wound microenvironment and support rapid wound healing.

### 2.6. Cytotoxicity Assay

**Figure 5:**
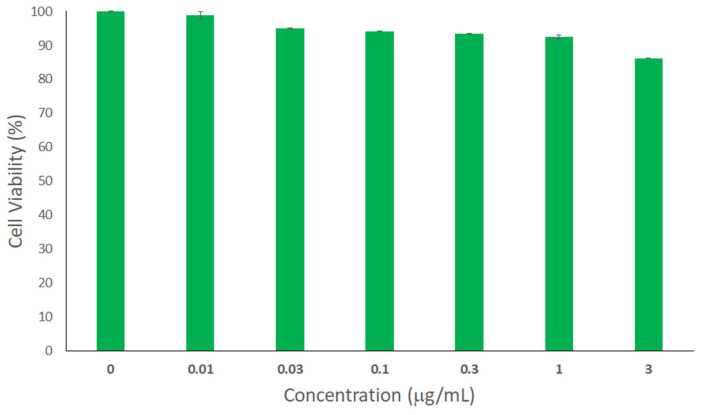
Evaluation of the cytotoxicity of the optimal H/CS/PLGA NPs at various concentrations ranging from 0.01 to 3 µg/mL using the SRB assay against human skin fibroblast cells. Untreated cells were used as negative control and considered to have 100% viability. No statistically significant difference in cell viability was observed for H/CS/PLGA NPs compared to the control (*p* < 0.05). All experiments were conducted in triplicates, and the mean values were calculated. Error bars represent ± standard deviation.

Biocompatibility with wound tissues is an important parameter that should be considered while developing wound healing products. In this regard, human skin fibroblasts were treated with different concentrations of H/CS/PLGA NPs. The cell viability of noncancerous cells was tested using the Sulforhodamine B Colorimetric (SRB) assay. The % cell viabilities of all concentrations were above 85% (Figure 5), with no significant difference compared to the control. These findings demonstrate the biocompatibility of the designed H/CS/PLGA NPs with skin fibroblast cells [43].

### 2.7. In Vitro Scratch Wound Healing Assay

**Figure 6:**
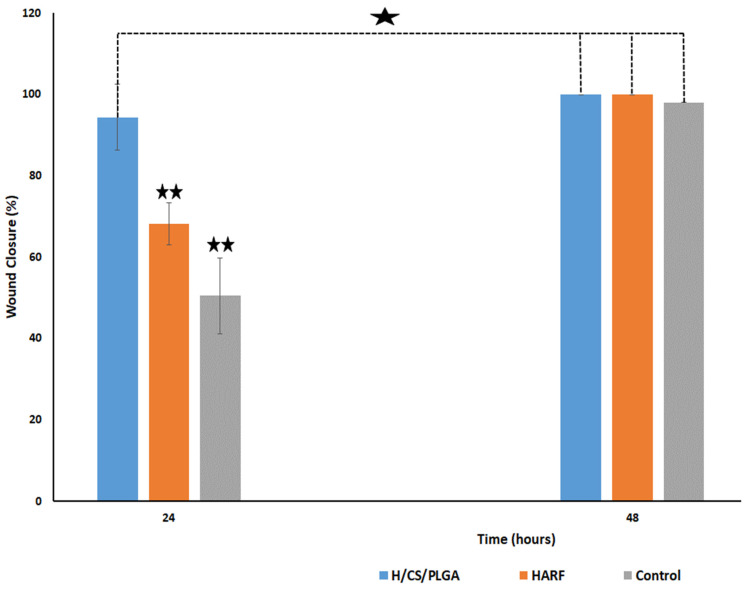
Wound closure percentage obtained after using HARF, blank formulation, and optimized H/CS/PLGA NPs, averaged from triplicate. One star indicates a non-significant difference, while a double star indicates a significant difference at *p* < 0.01.

The scratch wound healing assay was used to study the in vitro wound healing capabilities using human skin fibroblast cells. This assay was performed to observe the effect of the optimized H/CS/PLGA NPs on the healing process, compared to free HARF, and a blank CS/PLGA NPs formulation was used as a control. Wound closure percentages (averaged from triplicate) after 24, 48, and 72 h are presented in Figure 6 and Figure S1. The wound closure percentage result after 24 h treatment with H/CS/PLGA NPs was 94.40%, significantly higher than after the application of HARF alone (68.20%) or the blank formulation (50.50%), *p* < 0.01. In addition, the results showed a non-significant difference in wound closure percentage after 48 h, with a wound closure percentage of about 100%. Interestingly, it was also shown that there was a non-significant difference between the wound closure percentage after 24 h of H/CS/PLGA and 48 h of application of either HARF alone or the control. This may be attributed to the enhanced entrapment of HARF after loading into CS/PLGA NPs. The promising wound-healing ability of *P. harmala* alkaloids was reported, and their capacity to decrease the wound epithelial gap and increase the collagen and fibroblasts in the wound microenvironment, resulting in a rapid and effective healing process was shown [10]. Furthermore, CS was reported to promote wound healing by enhancing the migration of fibroblasts and collagen deposition in the wound area [21,22,23]. At the same time, PLGA is a wound-healing agent because it is a source of lactate that accelerates new vascularization, activates procollagen factors, and improves the recruitment of endothelial progenitor cells to the wound, and hence, promotes wound healing. On the other hand, acids such as boric acid, acetic acid, and ascorbic acid have been reported for the treatment of wound infections. The acidic environment imparted by lactate enhances antimicrobial activity, influences protease activity, reduces the toxicity of bacterial products, and enhances epithelization [26,27].

These findings shed more light on the importance of using HARF-loaded CS/PLGA NPs as a nanoformulation for effective wound healing.

## 3. Conclusions

The optimized H/CS/PLGA NPs were prepared and showed an average particle size of 202.27 ± 2.44 nm, a PDI of 0.23 ± 0.01, a zeta potential of 9.22 ± 0.94 mV, and an entrapment efficiency of 86.77 ± 4.18%. The optimal H/CS/PLGA NPs exhibited acceptable cytotoxicity and enhanced antimicrobial and wound healing properties compared to free HARF or CS/PLGA NPs. The designed H/CS/PLGA NPs revealed the highest antibacterial activity against *Staphylococcus aureus* and *Escherichia coli*, with MIC values of 0.125 and 0.06 mg/mL, respectively, compared to free HARF (MIC of 0.50 mg/mL). The prepared nanoparticles were found to be biocompatible when tested on human skin fibroblasts. Moreover, the wound closure percentage after 24 h of applying H/CS/PLGA NPs was 94.4 ± 80%, compared to free HARF and CS/PLGA NPs (68.20 ± 5.10 and 50.5 ± 9.40%; respectively). Thus, the developed H/CS/PLGA NPs exhibited enhanced antibacterial and wound healing activities and represent an effective biocompatible wound healing formulation.

## 4. Materials and Methods

### 4.1. Materials

Low molecular weight chitosan (CS) and poly(d,l-lactide-co-glycolide) (PLGA) were purchased from Sigma Aldrich (St. Louis, MO, USA). Polyvinyl alcohol (PVA; 98% hydrolyzed, MW~13,000) was purchased from Acros Organics (Geel, Belgium). Dimethylsulfoxide and acetic acid glacial were purchased from Fisher Chemicals (Fair Lawn, NJ, USA). Phosphate buffered saline was purchased from Lonza (Basel, Switzerland) and Tween 80 was purchased from El-Nasr Pharmaceutical Chemicals Co. (Cairo, Egypt). Tryptic Soy Agar (cat no. 10548) was purchased from Millipore (Bedford, MA, USA). Streptomycin, penicillin, fetal bovine serum, trichloroacetic acid (TCA), Dulbecco’s Modified Eagle’s Medium (DMEM) SRB, and tris(hydroxymethyl)aminomethane (TRIS) were purchased from Lonza (Basel, Switzerland).

### 4.2. Plant Material

Dried mature seeds of *P. harmala* L. were purchased from the local Egyptian market. A voucher specimen was deposited (18.1.17) at the Department of Pharmacognosy Herbarium, Faculty of Pharmacy, Cairo University (Cairo, Egypt).

### 4.3. Methods

#### 4.3.1. Extraction and Isolation of Major *P. harmala* Alkaloids

The extraction, isolation, and identification of the major alkaloids of *P. harmala* seeds were conducted as previously reported [8,9].

#### 4.3.2. Experimental Design

Statistical optimization of the variables of CS/PLGA NPs, loaded with HARF, was carried out using a 3^3^ (i.e., three-factor, three-level) Box–Behnken response surface design (BBD). The experimental design was constructed and assessed employing Design-Expert^®^ software (Version 12, Stat-Ease Inc., Minneapolis, MN, USA). The three independent variables were the HARF:PLGA weight ratio (A), the CS: PLGA weight ratio (B), and the sonication time (C). As presented in Table 2, the levels of these factors were chosen as (−1, 0, and +1). On the other hand, the responses (dependent variables) were selected as average particle size (Y1), polydispersity index (Y2), zeta potential (Y3), and entrapment efficiency (Y4).

The ranges of the low and high levels for each independent parameter were chosen based on preliminary experiments conducted to obtain an optimized H/CS/PLGA NPs. The selection of low and high levels of the three factors was made based on their effect on the nominated dependent variables. Then, based on these two levels, the medium levels of the three factors were created automatically by the software. Based on the followed BBD, with five center point per block repetitions, 17 experimental runs were performed to prepare the formulations. The 17 runs of the experimental design with their compositions and sonication time are presented in Table S2. Analysis of variance (ANOVA) was used to assess the model and term significance at *p* < 0.05.

#### 4.3.3. Preparation of H/CS/PLGA NPs

H/CS/PLGA NPs were prepared using the oil/water emulsion-solvent evaporation technique as described elsewhere [5,44] with some modifications.

The NPs were prepared by employing a SONOREX DIGITAL 10 P bath sonicator (BANDELIN electronic GmbH & Co. KG, Berlin, Germany) at different time intervals (4, 8, and 12 min). The emulsification step was performed using a bath sonicator to avoid any probable source of contamination when using either probe sonicators or homogenizers [44]. Briefly, the aqueous phase was prepared by the addition of polyvinyl alcohol (acting as a surfactant) to 1% (*v*/*v*) acetic acid in distilled water to obtain a 1% (*w*/*v*) PVA aqueous solution. To the prepared solution, chitosan was added to reach different final *w*:*w* ratios of chitosan: PLGA (0:1, 0.4:1, and 0.8:1). The emulsification step involved the subsequent dropwise addition of 0.5 mL DMSO containing 4 mg of PLGA and different *w*:*w* ratios of HARF:PLGA (0.1:1, 0.3:1, and 0.5:1) to 10 mL of the previously prepared aqueous solution. The resulting oil/water nano-emulsion was stirred continuously on a magnetic stirrer at its minimum speed and allowed to evaporate overnight. Part of this colloid was kept for particle size and zeta potential determination, while the rest of the solution was lyophilized for 72 h using a freeze dryer (TOPTION TOPT-10C Freeze dryer, Toption Group Co., Limited, Xi’an, China) after the addition of 2% (*w*/*v*) mannitol as a cryoprotectant. The dried NPs extract was stored at room temperature in a desiccator for further experiments.

#### 4.3.4. Average Particle Size, Polydispersity Index (PDI), and Zeta Potential Measurements

The average particle size and PDI of all the H/CS/PLGA NPs samples were determined using dynamic light scattering, employing a Zetasizer Nano ZS (Malvern Instruments, Herrenberg, Germany) [45]. All measurements took place at 25 °C, where the refractive index and viscosity of the water were 1.33 and 0.887 mPa.s, respectively. The instrument was equipped with a 10 mW HeNe laser, allowing for the measurements to be performed at a wavelength of 633 nm and a detection angle of 173° backscatter. The zeta potential of all samples was measured by employing laser Doppler velocimetry (Malvern Instruments, Herrenberg, Germany) in a clear disposable folded capillary cell (DTS1070, Malvern Instruments). All measurements took place in triplicates, and standard deviation (SD) was calculated.

#### 4.3.5. Determination of Entrapment Efficiency (EE%)

Direct determination of the EE % of all H/CS/PLGA/NPs samples was carried out as previously described by Nair et al., 2012 [46], with some modifications. Briefly, 2 mL of H/CS/PLGA/NPs were centrifuged for 1 h at 5000 rpm (Hermle Z 326 K, Labortechnik GmbH, Wehingen, Germany). Then, the supernatant was discarded, and the NPs were dispersed in 1:1 DMSO:1% acetic acid in distilled water; this solvent mixture was selected to dissolve both the chitosan coat and the PLGA NPs. After complete dissolution via vigorous vortexing, the UV absorbance of the clear solution was determined using a CARY 500 UV–vis–NIR Scan dual-beam spectrophotometer (Varian, Palo Alto, CA, USA). Then, the total amount of HARF loaded in the NPs was estimated from the equation generated from the calibration curve. The EE % of the H/CS/PLGA/NPs was determined using Equation (1).

(1) $$\mathrm{EE}\;\%=\frac{\mathrm{Total}\;\mathrm{amopunt}\;\mathrm{of}\;\mathrm{loaded}\;\mathrm{HARF}}{\mathrm{Theoretical}\;\mathrm{HARF}\;\mathrm{loading}}\times 100$$

#### 4.3.6. Formulation Optimization

Design-Expert 12^®^ software was used to determine the optimum H/CS/PLGA NPs formulation. The specific constraints applied on the average particle size, PDI, zeta potential, and EE% are presented in Table S2. To confirm the validity of the generated predicted factors and responses, the developed optimum formulation was prepared and characterized. In this regard, three prediction points were performed, and the software averaged the response data. For further investigations, the optimum formulation was prepared, as described in Section 4.3.3., and lyophilized (at −40 °C) for 72 h using a freeze dryer (TOPTION TOPT Freeze dryer, Toption Group Co., Limited, Xi’an, China).

#### 4.3.7. Characterization of the Optimal H/CS/PLGA NPs Formulation

The NPs’ morphology and size were studied using transmission electron microscopy (TEM) using a JEOL-JEM 2100 electron microscope (Musashino, Akishima, Tokyo, Japan) operating at 160 kV. Deionized water was used to dilute a 50 µL aliquot of the NPs at a ratio of 2:1 (*v*/*v*). Then, the diluted NPs were stained with 2% aqueous phosphotungstic acid. This mixture was added dropwise and dried over a carbon-coated copper 200 mesh grid, imaged, and photographed. The average particle size (nm) histogram of the NPs was produced using the image processing program ImageJ (NIH, Bethesda, MD, USA) [8,9].

The FT-IR spectra of HARF, blank CS/PLGA NPs, and the optimal H/CS/PLGA NPs formulation were obtained using Attenuated Total Reflection Fourier-Transformed Infrared Spectroscopy (ATR-FTIR) involving a Nicolet 380 spectrometer (ThermoScientific Nicolet, Waltham, MA, USA), and the spectra were recorded in the range of 4000–500 cm^−1^. [8]

#### 4.3.8. In Vitro Release Study of HARF from the Optimal H/CS/PLGA NPs Formulation

The in-vitro drug release test was carried out as previously described by Hammam et al., 2017 [47], with some modifications. Briefly, an amount of the lyophilized H/CS/PLGA NPs was resuspended in phosphate buffer saline (with added 2% tween 80; pH 7.4). The prepared colloids were incubated at 37 °C while shaking at 100 rpm. After gentle mixing of the colloids, samples were withdrawn at specific time intervals. Then, the samples were centrifuged (model Z 326 K, Hermle, Labortechnik GmbH, Wehingen, Germany) at 15,000 rpm for 30 min, and the supernatant was saved for drug concentration analysis. UV absorbance analysis at 259 nm was performed using a FLUOstar Omega microplate reader (BMG Labtech, Offenburg, Germany). All experiments were performed in triplicates, and the standard deviation (SD) was calculated.

#### 4.3.9. Antimicrobial Assay for the Optimal H/CS/PLGA NPs Formulation

##### Inoculum Preparation (Colony Suspension Method)

*Staphylococcus aureus* ATCC^®^ 6538 (Lot no. 4600502) and *Escherichia coli* ATCC^®^ 8739 (Lot no. 380063) were obtained from American Type Culture Collection (University Boulevard, Manassas, VA, USA). The bacterial strains were inoculated separately into 100 mL of Tryptic Soy Broth medium and then incubated at 37.0 ± 1.0 °C for 24 ± 2 h. A loopful from the broth was streaked onto Tryptic Soy Agar medium, and incubated at 37.0 °C for 21 ± 3 h to prepare a fresh culture agar plate. A direct sterile saline solution was prepared by inoculating 3–4 colonies (from each organism plate), then the suspension was adjusted to achieve turbidity that was equivalent to a 0.5 McFarland standard. The inoculum density was standardized using a 0.5 McFarland standard and DensiCHEK© optical device (DensiCHEK plus© SKU Number: 21250; BioMérieux, France). Suspensions containing approximately 1.0 × 10^8^ CFU/mL of *Escherichia coli* and *Staphylococcus aureus* were obtained.

##### Broth Macrodilution Method

To test the antimicrobial activities, 5.0 mL from free HARF, CS/PLGA blank NPs, and H/CS/PLGA NPs were added to 5.0 mL tube broth (1:2 dilution) and mixed well. Then, 5.0 mL was aspirated using a new tip and added to the next 5.0 mL broth (1:4). That step was repeated for the preparation of ten dilutions for each antimicrobial sample. Then, 1.0 mL from the sample was inoculated in the first well in the 24-well plate, and 1.0 mL from each dilution was consequently inoculated in the remaining wells. Afterward, 100 µL of inoculum prepared was added to each well. This resulted in a final concentration of 5.0 × 10^5^ CFU/mL. Another 100 µL from each organism suspension was diluted and cultured (externally) to confirm the inoculum density. A growth control well containing inoculated broth, without sample, was added to each organism/sample. All plates were incubated at 37.0 °C for 24 h. After incubation, plates were removed from the incubator and placed in the dark to check growth. All growth control wells yielded a turbid solution, indicating the validity of the test. Inoculum density culture results were confirmed to have a concentration of 4–6 × 10^5^ CFU/mL for all tested organisms, according to Clinical and Laboratory Standards Institute (CLSI) procedures (document M07) [48].

#### 4.3.10. Cytotoxicity Assay

##### Cell Culture

Human skin fibroblasts were obtained from the American Type Culture Collection, and maintained in DMEM supplemented with streptomycin (100 mg/mL), penicillin (100 units/mL), and 10% heat-inactivated fetal bovine serum. Cells were incubated in humidified 5% (*v*/*v*) CO_2_ at 37 °C.

##### Sulforhodamine B Colorimetric Assay

Human skin fibroblasts were treated with different concentrations of H/CS/PLGA NPs. The cell viability was tested using the SRB assay as described previously [8,9,15,16]. The percentage of cell viability was reported as the cytotoxicity results. All trials were conducted in triplicates, and data were displayed as mean ± standard deviation.

#### 4.3.11. In Vitro Scratch Wound Healing Assay

The scratch wound assay is based on generating a gap in a confluent monolayer of fibroblasts to simulate a wound. In this regard, human skin fibroblast cells were plated at a density of 3 × 10^5^/well onto a 6-well plate and cultured overnight in 5% FBS-DMEM at 37 °C and 5% CO_2_. The following day, mechanical scratches were introduced horizontally into the confluent monolayer, employing a sterile pipette tip. Then, the medium of each flask was discarded, and the cells were washed thoroughly with PBS at pH 7.4. Fresh media were then added, which contained either free HARF or H/CS/PLGA NPs. The untreated cells were used as a control and the incubation time was 48 h for all experiments. Images were taken using an inverted microscope at 0, 24, and 48 h. The plates were incubated at 37 °C and 5% CO_2_ in-between time points. The acquired images (Figure S1) were investigated using MII ImageView software version 3.7 (Informer technologies Inc., Los Angeles, CA, USA) [49,50,51,52,53,54], and the wound closure percentage was computed using Equation (2) as follows:

(2) $$\mathrm{Wound}\;\mathrm{Closure}\%=\left[\frac{\mathrm{At}=0h-\mathrm{At}=\Delta h}{\mathrm{At}=0h}\right]\times 100$$

where (At = 0 h) is the average area of the wound measured immediately after scratching (time zero), and (At = Δh) is the average area of the wound measured in h hours after the scratch was performed. The results are displayed as mean ± SD. One-way analysis of variance (ANOVA) was carried out, followed by a posthoc test, Duncan test, using the Statistical Package for Social Sciences (SPSS) version 25 (IBM SPSS, Inc., Chicago, IL, USA) at *p* < 0.01.

## Figures and Tables

**Table 1:** Model summary statistics of the different models for tested responses, constraints for optimization of H/CS/PLGA NPs formulation, and the predicted and observed values of the responses.

Response		Model	R^2^	Adjusted R^2^	Predicted R^2^	Constraints	Predicted	Observed	95% Prediction Interval
Y1	Particle Size (nm)	Quadratic model	0.96	0.90	0.61	In range	204.84	202.27 ± 2.44	198.56–211.13
Y2	PDI	Quadratic model	0.97	0.92	0.53	Minimize	0.10	0.23 ± 0.01	0.07–0.13
Y3	Zeta Potential (mV)	Linear model	0.91	0.89	0.87	Maximize	8.79	9.22 ± 0.94	7.12–10.45
Y4	Entrapment Efficiency (%)	2FI model	0.95	0.92	0.84	Maximize (lower limit: 80%)	89.50	86.77 ± 4.18	79.99–99.01

**Table 2:** Independent variables and their relevant levels of 3^3^ BBD for H/CS/PLGA NPs, and their levels relative to the optimized H/CS/PLGA NPs formulation.

Factors		Levels of Factors			Levels for the Optimized Formulation
		Low (−1)	Medium (0)	High (+1)	
A	HARF:PLGA weight ratio	0.1:1	0.3:1	0.5:1	0.10
B	CS:PLGA weight ratio	0	0.4:1	0.8:1	0.60
C	Sonication time (min)	4	8	12	12

**Table 3:** Antibacterial activity of optimal H/CS/PLGA NPs and free HARF against *Staphylococcus aureus and Escherichia coli*.

Bacterial Strain	Minimum Inhibitory Concentration (MIC in mg/mL)		
	Harmala Alkaloid Rich Fraction (HARF)	CS/PLGA NPs	H/CS/PLGA NPs
*Staphylococcus aureus*	0.5	0.18	0.13
*Escherichia coli*	0.5	0.18	0.06

## Supplementary Materials

The following are available online at https://www.mdpi.com/article/10.3390/nano11092438/s1, Table S1: Average particle size, polydispersity index, zeta potential, and entrapment efficiency % of the prepared H/CS/PLGA NPs formulations, Table S2: Composition and sonication time of the 3^3^ BBD for H/CS/PLGA NPs, Figure S1: Representing images for the in vitro scratch wound healing assay using human skin fibroblasts cultured on polystyrene plates for (A) H/CS/PLGA NPs, (B) free HARF, and (C) CS/PLGA blank NPs. Images were taken using an inverted microscope at 0, 24, and 48 h. The experiment has been conducted in triplicates. Vertical black lines in all panels indicate scratch size.
