# Supplementary material for: Chitosan-Coated PLGA Nanoparticles Loaded with Peganum harmala Alkaloids with Promising Antibacterial and Wound Healing Activities
Source: Nanomaterials (Basel). 2021 Sep 18;11(9):2438. doi: 10.3390/nano11092438 (PMC8464825; doi:10.3390/nano11092438)
Supplement: Supplementary file 1 [file nanomaterials-11-02438-s001.zip › nanomaterials-1360636-supplementary.pdf]

Supplementary Material

Table S1. Average particle size, polydispersity index, zeta potential, and entrapment efficiency % of the prepared H/CS/PLGA NPs formulations. 50.29 ±2.38

Formula	Particle Size ±SD (nm)	PDI ±SD	Zeta Potential ±SD (mV)	Entrapment Efficiency ±SD (%)
**F1**	188.2 ± 5.66	0.11 ±0.03	12.33 ±1.55	50.29 ± 2.38
**F2**	205.8 ± 9.79	0.08 ±0.04	9.1 ±0.79	46.25 ± 7.97
**F3**	205.25 ± 1.34	0.10 ±0.03	11.5 ±6.15	60.03 ± 4.62
**F4**	197.23 ± 2.57	0.12 ± 0.005	6.24 ± 0.37	48.91 ± 5.59
**F5**	210.35 ± 1.48	0.15 ± 0.04	10.65 ± 0.92	87.84 ± 16.00
**F6**	202.93 ± 0.83	0.13 ± 0.02	7.42 ± 0.22	58.418 ± 5.48
**F7**	198.2 ± 5.80	0.13 ± 0.019	8.99 ± 2.53	53.84 ± 2.02
**F8**	197.47 ± 4.17	0.11 ± 0.023	5.34 ± 0.78	46.89 ± 10.36
**F9**	197.6 ± 3.39	0.12 ± 0.03	9.16 ± 1.11	51.42 ± 3.34
**F10**	205.27 ± 7.31	0.23 ± 0.095	8.50 ± 0.40	24.89 ± 2.97
**F11**	197.28 ± 12.88	0.14 ± 0.011	5.52 ± 3.56	45.90 ± 8.47
**F12**	203.65 ± 2.33	0.17 ± 0.018	3.45 ± 0.28	32.97 ± 2.07

**F13**	214.10 ± 4.81	0.18 ± 0.016	12.2 ±2.12	32.47 ±0.03
**F14**	202.80 ± 4.86	0.25 ± 0.13	9.27 ±2.73	50.58 ±7.72
**F15**	199.42 ± 11.63	0.13 ± 0.057	9.22 ±1.24	54.07 ±0.05
**F16**	198.20 ± 8.34	0.12 ± 0.027	7.39 ±6.6	75.28 ±6.82
**F17**	180.12 ± 2.04	0.11 ± 0.012	2.63 ±0.42	44.74 ±4.84

Table S2. Composition and sonication time of the 3^3 BBD for H/CS/PLGA NPs.

Formula	HARF : PLGA weight ratio	CS: PLGA weight ratio	Sonication time (min)
F1	0.3	0.8	4
F2	0.5	0.4	4
F3	0.3	0.8	12
F4	0.3	0.4	8
F5	0.1	0.8	8
F6	0.3	0.4	8
F7	0.3	0.4	8
F8	0.5	0	8
F9	0.3	0.4	8
F10	0.5	0.4	12
F11	0.3	0	4
F12	0.1	0	8
F13	0.5	0.8	8
F14	0.1	0.4	4
F15	0.3	0.4	8
F16	0.1	0.4	12
F17	0.3	0	12

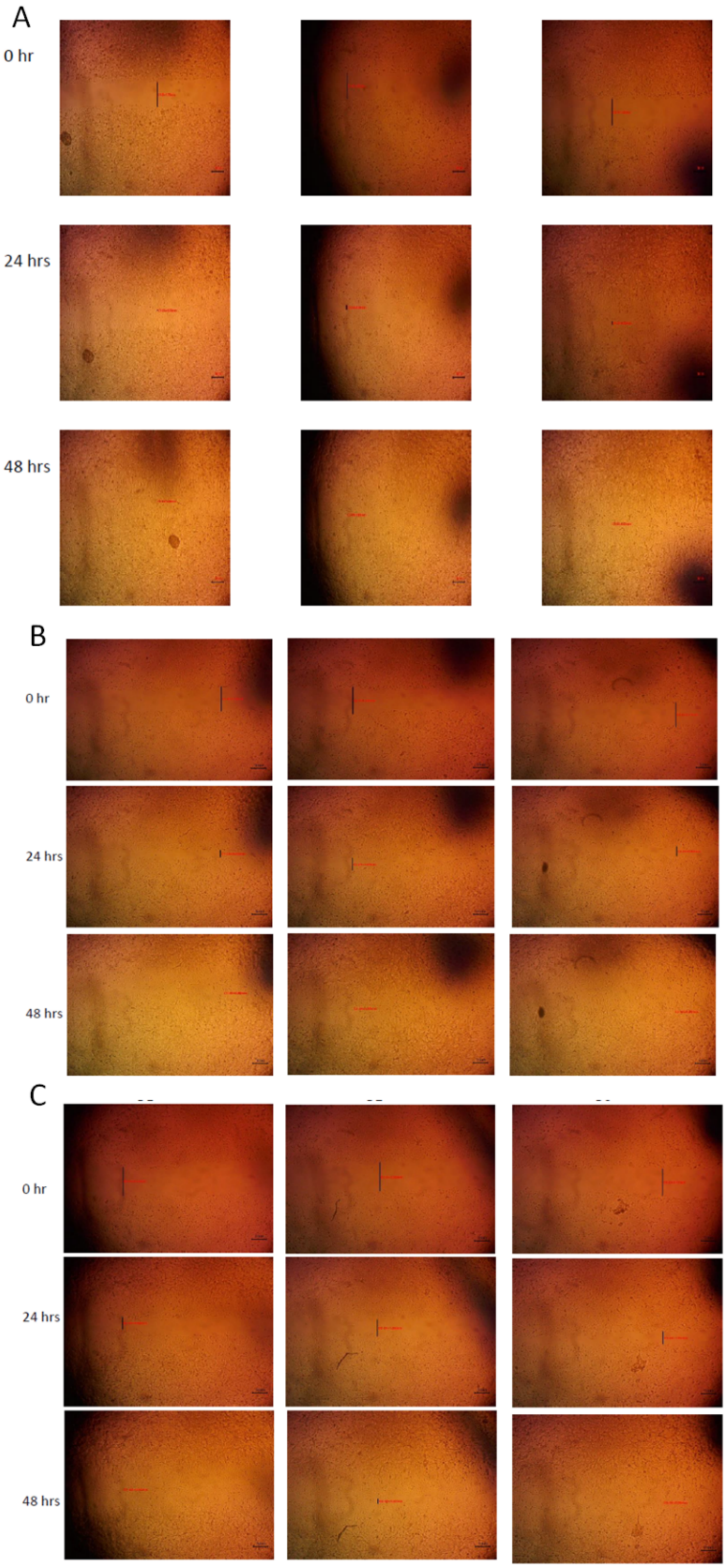

Figure S1. Representing images for the *in vitro* scratch wound healing assay using human skin fibroblasts cultured on polystyrene plates for (**A**) H/CS/PLGA NPs, (**B**) free HARF, and (**C**) CS/PLGA blank NPs. Images were taken using an inverted microscope at 0, 24, and 48 h. The experiment has been conducted in triplicates. Vertical black lines in all panels indicate scratch size.
